# MicroRNA-30e regulates neuroinflammation in MPTP model of Parkinson’s disease by targeting Nlrp3

**DOI:** 10.1007/s13577-017-0187-5

**Published:** 2017-12-22

**Authors:** Dongsheng Li, Hongqi Yang, Jianjun Ma, Sha Luo, Siyuan Chen, Qi Gu

**Affiliations:** Department of Neurology, Henan Provincial People Hospital, No. 7 Weiwu Road, Jinshui District, Zhengzhou, 450003 Henan China

**Keywords:** Parkinson’s disease, Neuroinflammation, Neurodegeneration, Nlrp3 inflammasome, miR-30e

## Abstract

Accumulating evidences suggest that neuroinflammation is a pathological hallmark of Parkinson’s disease (PD), a neurodegenerative disorder characterized by loss of dopaminergic neurons in substantia nigra pars compacta (SNpc). MicroRNAs have been recently recognized as crucial regulators of inflammatory responses. Here, we found significant downregulation of microRNA-30e (miR-30e) in SNpc of MPTP-induced PD mice. Next, we employed miR-30e agomir to upregulate miR-30e expression in MPTP-treated mice. Our results showed that delivery of miR-30e agomir remarkably improved motor behavioral deficits and neuronal activity, and inhibited the loss of dopamine neurons. Moreover, the increased α-synuclein protein expression in SNpc of MPTP-PD mice was alleviated by the upregulation of miR-30e. Further, miR-30e agomir administration also attenuated the marked increase of inflammatory cytokines, such as TNF-α, COX-2, iNOS, and restored the decreased secretion of BDNF in SNpc. In addition, we demonstrated for the first time that miR-30e directly targeted to Nlrp3, thus suppressing Nlrp3 mRNA and protein expression. Finally, miR-30e upregulation significantly inhibited the activation of Nlrp3 inflammasome as evident from the decreased Nlrp3, Caspase-1 and ASC expressions and IL-18 and IL-1β secretions. Taken together, our study demonstrates that miR-30e ameliorates neuroinflammation in the MPTP model of PD by decreasing Nlrp3 inflammasome activity. These findings suggesting that miR30e may be a key inflammation-mediated molecule that could be a potential target for PD therapeutics.

**Electronic supplementary material:**

The online version of this article (doi:10.1007/s13577-017-0187-5) contains supplementary material, which is available to authorized users.

## Introduction

Parkinson’s disease (PD) is the second only to Alzheimer’s disease (AD) as the most common neurodegenerative movement disorder, which is characterized with progressive loss of dopaminergic neurons in the substantia nigra pars compacta (SNpc) and accumulation of α-synuclein (α-syn) in Lewy bodies [1, 2]. The clinically used drugs, including L-DOPA, monoamine oxidase type B inhibitors and catechol-O-methyltransferase inhibitors, could only ameliorate the symptoms through supplementing the absent dopamine, but fail in delaying the process of dopamine neuronal degeneration because it could not protect against neurons injury [3–5]. Thus, developing a more effective agent remains the top priority in prevention and treatment of PD.

Several lines of researches have suggested that neuroinflammation is considered as the major central event in the process of dopaminergic neuronal cell death in PD [6–8]. Enhanced levels of proinflammatory cytokines such as TNF-α, COX-2, IL-1β and IL-18 can be found in the analysis of postmortem brain of PD patients [9, 10]. Moreover, the activity of IL-1β and IL-18 is critical controlled by a cytoplasmic multiprotein, called “inflammasome”, which contains nod-like receptor protein 3 (Nlrp3), adaptor protein ASC and proinflammatory mediators Caspase-1, IL-18 and IL-1β [11]. Activation of Nlrp3 inflammasome has been observed in a variety of neurodegenerative diseases, including AD and amyotrophic lateral sclerosis (ALS) [12]. Importantly, Nlrp3 might be associated with the development of PD and be a potential target for the treatment of PD [6, 13]. However, the mechanisms underlying the regulation of Nlrp3 inflammasome activity in PD are poorly understood.

Accumulating evidences indicate that post-transcriptional regulation by microRNAs (miRs) is important for the regulation of gene expression and inflammatory responses [6, 14]. Sustained aberrant expression levels of several different miRs have been described in inflammation-related neurodegenerative disorders, including multiple sclerosis (MS), AD, ALS and PD [15–18]. Therefore, identification of novel miR machinery that modulates neuroinflammation not only helps to understand the development of PD, but also provides a new approach for the treatment of PD. In our study, we found that exogenous delivery of miR-30e ameliorated neuronal injury, neuroinflammaiton and dyskinesia in MPTP-induced PD mice. Furthermore, miR-30e directly targeted to Nlrp3, which in turn mediated Nlrp3 inflammsome activity and inflammation.

## Materials and methods

### Materials and reagents

8-week-old male C57BL/6 mice were purchased from the SLAC Laboratory (Shanghai, China) and were maintained in cages with constant temperature (21 ± 1 °C), relative humidity (60%), a strict 12 h/12 h light–dark cycle, and free access to water and food. The experimental protocol was approved by Institutional Animal Care and Use Committee of Henan Provincial People Hospital and carried out in accordance with the guidelines for the Care and Use of Laboratory Animals.

MiR-30e agomir, miR-30e mimics, and corresponding negative control miRNA were obtained from GenePharm (Shanghai, China). SuperScriptIII First-Strand Synthesis system, fetal bovine serum (FBS) Dulbecco’s modified Eagle’s medium, penicillin, streptomycin and lipofectamine 2000 were purchased from Invitrogen (CA, USA). Antibodies against α-syn, Nlrp3, ASC, Caspase-1 and β-actin were from Cell Signaling Technology (MA, USA). Tyrosine hydroxylase (TH) antibody, horseradish peroxidase (HRP)-conjugated goat anti-rabbit IgG antibody, HRP-donkey anti-goat IgG antibody and the Enhanced Chemiluminesecence Kit were from Millipore (MA, USA). Nissl staining solution, diaminobenzidine (DAB) and RIPA buffer were purchased from Beyotime (Jiangsu, China). All chemicals and reagents unless otherwise indicated were obtained from Sigma (MO, USA).

### Animal model and miR-30e agomir delivery

The mice were received 3 times of intraperitoneally (i.p.) injection of MPTP (20 mg/kg) at days 1, 7, and 14. Control mice were administrated with saline only. Mice were killed at different times after the first MPTP injection: 1, 3, 7, 10, and 14 days. For the delivery of miR-30e in MPTP mice, a stereotactic catheter was surgically implanted into the right lateral ventricle of mice (Bregma: −2 mm, Lateral: 2 mm, Dorsoventral: 3 mm). 5 μL of saline containing 20 nmol/L of miR-30e agomir or a scramble sequence control miRNA (negative control) was injected through the catheter per day for 7 consecutive days. The first treatment of agomir was performed 2 h after the last injection of MPTP. The schematic diagram of miR-30e administration is illustrated in Figure S1. Mice were killed immediately after behavioral assessments on day 21 by decapitation. The ventral midbrain containing the SNpc was dissected and stored at −80 °C for further experiments.

### Quantitative reverse transcription (qRT-PCR) analysis

Total RNA from SNpc tissues or BV-2 cells were extracted using RNAiso Plus Reagent (Takara, Dalian, China) and reverse transcription was performed with the SuperScriptIII First-Strand Synthesis system. Quantitative assay of genes expressions was performed using a SYBR QPCR Kit (Toyobo, Osaka, Japan) and ABI 7500 real-time PCR system (Applied Biosystems, CA, USA). The gene expression was normalized to the GAPDH and calculated using the ΔC_T_ method. The specific primer sequences used were listed in Table S1.

### Rota-rod test

The mice were evaluated for motor balance and coordination using a rotary rod apparatus (Harvard Apparatus, MA, USA) at different times as indicated. All animals were pretrained before staring the experiment. Each mouse was placed in the apparatus (diameter: 7 cm, length: 30 cm) and operates at a constant speed of 30 rpm. The three latencies to fall recorded by magnetic trip plates were averaged to yield a final value, and the maximum cutoff time was set as 180 s.

### Pole test

A wooden pole of ~ 50 cm in length and ~ 1 cm in diameter was wrapped in gauze and a cork ball of 2.5 cm was glued on top of the pole. Each mouse was placed on top of the ball and the time required for the mouse to climb down the pole was recorded. The test was repeated three times to evaluate the average. The cutoff time was 250 s.

### Traction test

The mice were suspended by their forepaws to a horizontal wire. The mouse was scored as 3 if grasped the wire with two hind paws, 2 if grasped the wire with one hind paw, and 1 if grasped the wire with any of the hind paws.

### Beam-crossing task

Each mouse was placed at one end of a 100 cm long and 2 cm wide beam, which was elevated 1 cm above the ground. The time required for the mouse to cross the beam was measured. The cutoff time was set as 120 s.

### Nissl staining

The midbrain tissues were fixed in 4% paraformaldehyde, embedded in paraffin, and cut into 4 μm thick sections. For Nissl staining, the sections were incubated with nissl staining solution at 50 °C for 20 min. After rinsing with distilled water, sections were dehydrated with 95% ethyl alcohol, 70% ethyl alcohol in secession. The number of staining cells in SNpc was counted using a BX51 light microscope (Olympus, Tokyo, Japan) at higher magnification (× 400).

### Immunocytochemistry

Sections of brain tissues were permeabilized with Triton X-100 and blocking with 1% goat serum in saline at room temperature, and incubated with a primary antibody to TH at 1:200 dilution at 4 °C overnight. After washing with saline, sections were incubated with secondary goat–rabbit IgG antibody for 1 h at room temperature and washed three times. DAB solution was added to incubate for 3 min. Images were captured with a BX51 light microscope.

### Western blotting

Total protein was extracted from selected mouse midbrain or BV-2 cells using RIPA lysis buffer, and quantified using a bicinchoninic acid protein assay kit (Thermo, MA, USA). Western blotting was performed as previously described [17]. Different primary antibodies used were as following: TH, α-syn, β-actin, ASC (diluted 1:1000), Nlrp3 and Caspase-1 (diluted 1:500). After incubation with corresponding secondary antibody (HRP-conjugated goat anti-rabbit or donkey anti-goat IgG antibody, diluted 1:1000), bands were visualized with the Enhanced Chemiluminescence Kit and determined with a densitometry software (Image J, NIH, Maryland, USA).

### Enzyme-linked immunosorbent assay (ELISA)

The level of TNF-α, COX-2, iNOS, BDNF, IL-18 and IL-1β in SNpc was determined using immunoassay kits with the instructions provided by manufacturer (R&D System, MN, USA). All samples were assayed and absorbance was read using a microplate reader (Multiskan Spectrum, Thermo, MA, USA).

### Cell culture

Murine BV-2 microglial cells were obtained from the Cell Bank of Chinese Academy of Medical Science (Shanghai, China) and were maintained in Dulbecco’s modified Eagle’s medium with 10% heat-inactivated FBS, 100 U/mL penicillin and 100 mg/mL streptomycin at 37 °C in a humidified incubator under 5% CO_2_ condition.

### In vitro miR-30e mimics transfection

For overexpression of miR-30e in BV-2 cells, the cells were transfected with miR-30 mimics or negative control miRNA using Lipofectamine 2000 according to the manufacturer’s protocol. After 48-h transfection, cells lysis was used for luciferase assay or western blotting analysis.

### Luciferase reporter assay

The binding of miR-30e to the target gene Nlrp3 was assayed by luciferase experiment. A wild-type murine Nlrp3 mRNA 3′UTR luciferase reporter construct was amplified by PCR from the Nlrp3 mRNA (NM_145827) 3′-UTR sequence and then cloned into the psiCHECK2-3′UTR vector (Ambio Inc., TX, USA). For mutant construct of Nlrp3 3′UTR, deletion mutagenesis and fusion-PCR were performed. BV-2 cells were co-transfected with either wild-type or mutant Nlrp3 3′UTR, plus miR-30e mimics or negative control for 48 h. Luciferase activity was assessed using Dual-Luciferase Reporter Assay System (Promega, WI, USA) according to the manufacturer’s instructions.

### Statistical analysis

Data were presented as mean ± SEM. The statistical significance of differences between two groups was analyzed by one-way analysis of variance (ANOVA) or the unpaired two-tailed Student’s *t* test using SPSS 10.0 statistical software (SPSS Inc., IL, USA). *P* < 0.05 was considered to be statistically significant.

## Results

### MiR-30e was downregulated in SNpc of MPTP-PD mice model

**Fig. 1:**
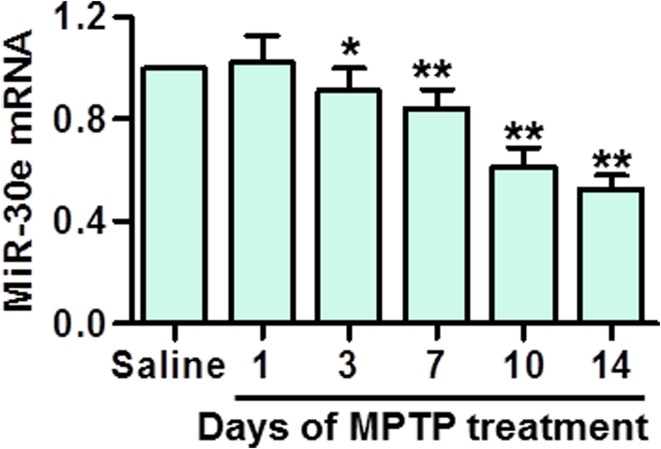
Decreased expression of miR-30e in SNpc after MPTP injection. Mice were killed at 1, 3, 7, 10, and 14 days after the first MPTP injection. The ventral midbrain containing the SNpc was dissected. The mRNA expression of miR-30e in SNpc was detected by qRT-PCR. *P < 0.05, **P < 0.01 vs. saline, *n* = 6 mice in each group

First, we examined the expression of miR-30e in SNpc of MPTP-treated mice by qRT-PCR. The results showed that the expression of miR-30e was significantly decreased after intraperitoneal injection of MPTP. At 3, 7, 10 and 14 days after the first MPTP injection, miR-30e expression was reduced to 0.91 ± 0.08-fold, 0.84 ± 0.07-fold, 0.61 ± 0.07, and 0.53 ± 0.06-fold of saline-treated mice, respectively (Fig. 1).

### Effect of miR-30e agomir on body weight in MPTP-administrated mice

**Fig. 2:**
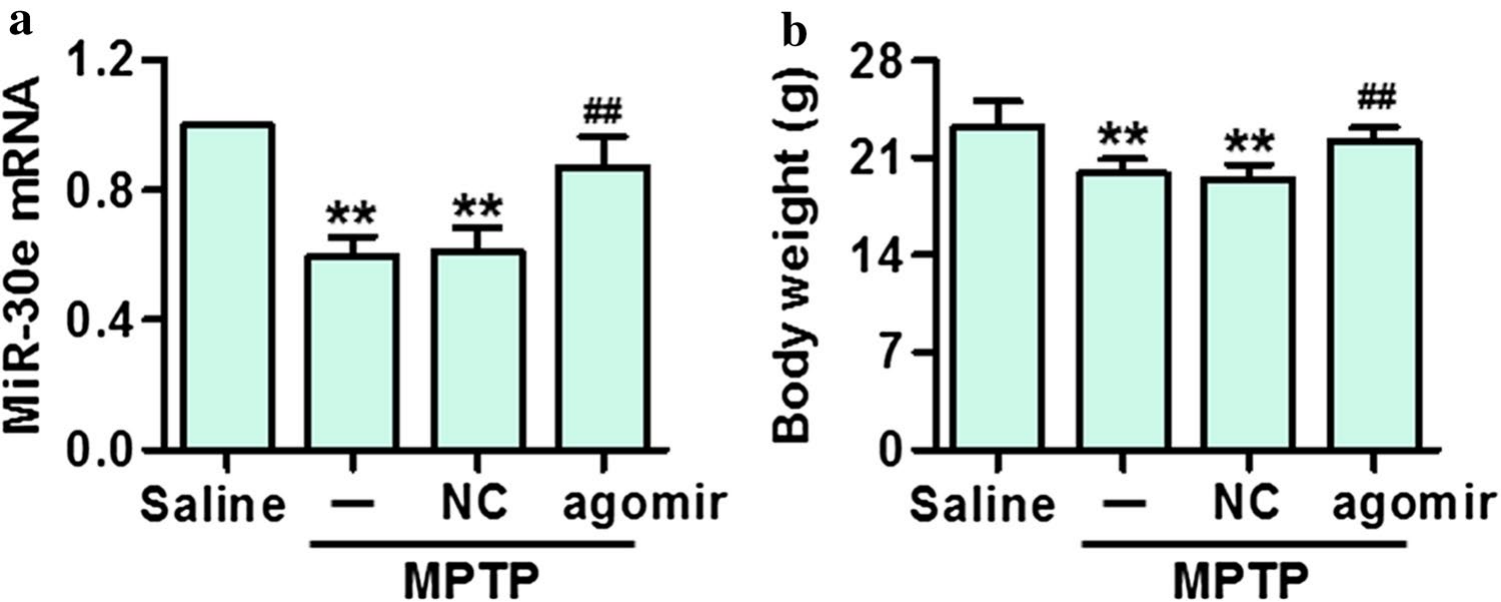
Effect of miR-30e on body weight in MPTP-administrated mice. **a** Mice were killed on day 21 by decapitation. The mRNA expression of miR-30e in SNpc was determined by qRT-PCR. **P < 0.01 vs. saline; ##*P* < 0.01 vs. MPTP, *n* = 6 mice in each group. **b** MiR-30e upregulation attenuated the decrease of body weight in MPTP-PD mice. **P < 0.01 vs. saline; ##*P* < 0.01 vs. MPTP, *n* = 18 mice in each group

To investigate the role of miR-30e in MPTP-PD mice, miR-30e agomir or negative control was injected into the right lateral ventricle of MPTP-PD mice to restore the expression of miR-30e. Expectedly, the expression of miR-30e in SNpc was markedly higher in miR-30e agomir-treated mice than in negative control-treated mice at 21 days after the first MPTP injection (Fig. 2a). Intraperitoneal injection of MPTP dramatically decreased body weight of mice compared to saline group. However, miR-30e agomir significantly improved body weight on day 21 in MPTP-administrated mice (Fig. 2b).

### MiR-30e upregulation improved the dyskinesia induced by MPTP

**Fig. 3:**
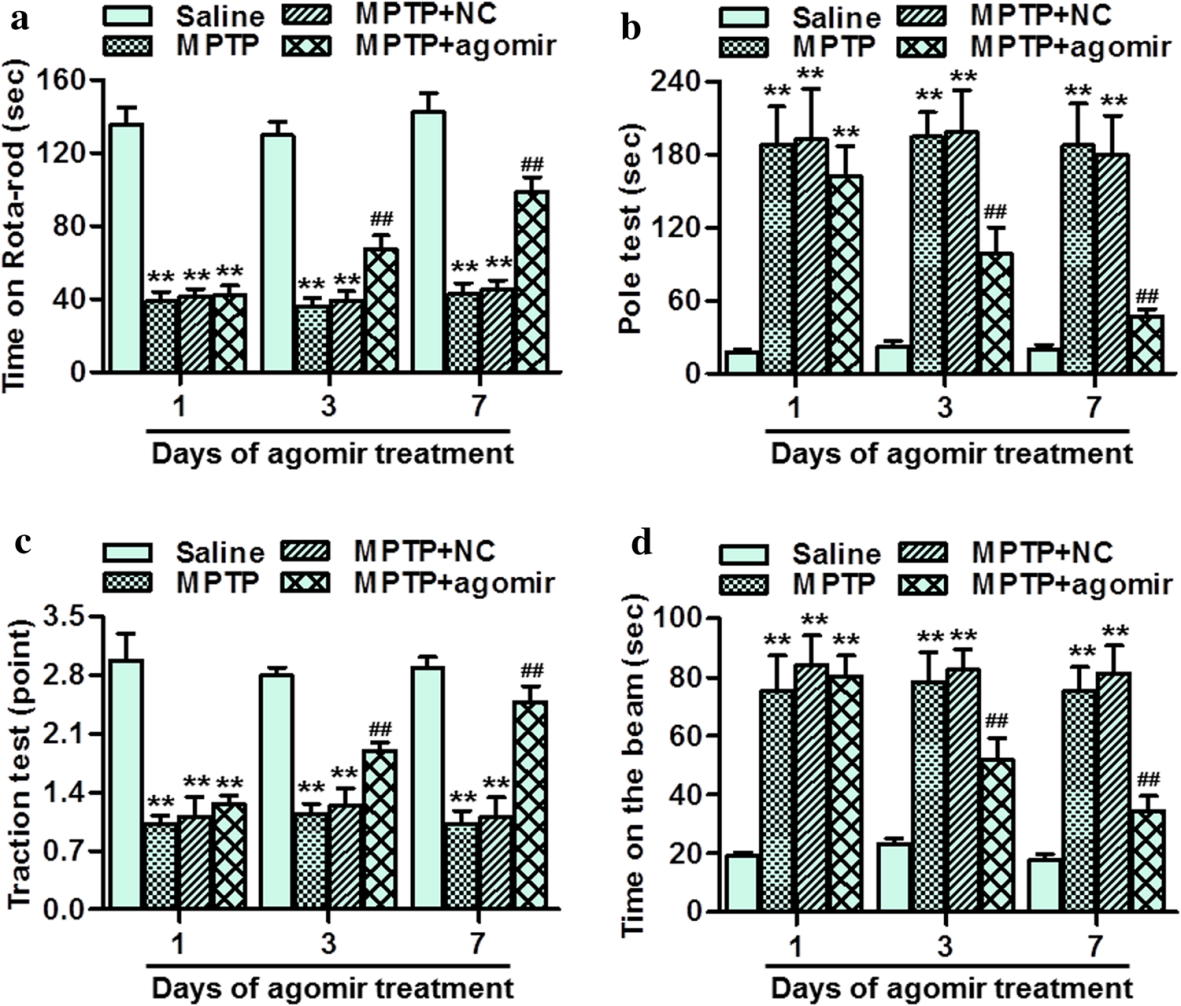
MiR-30e upregulation improved the dyskinesia in MPTP-PD mice. **a**–**d** Effect of miR-30e restoration on rota-rod test (**a**), pole test (**b**), traction test (**c**), and beam-crossing task (**d**) at 1, 3 and 7 days after the first miR-30e agomir treatment, respectively. **P < 0.01 vs. saline; ##*P* < 0.01 vs. MPTP, *n* = 12–16 mice in each group

To investigate the effect of miR-30e restoration on motor function, several kinds of behavior tests, including rota-rod test, pole test, traction test and beam-crossing task were conducted in the present study. The results of rota-rod test showed that MPTP injection significantly decreased rota-rod activity as compared to saline group. However, treatment with miR-30e agomir for 3 and 7 days showed significant improvement in rota-rod activity (Fig. 3a). Pole test showed that total locomotor activity was markedly increased at 1st, 3rd and 7th days after the last MPTP injection, which was significantly inhibited at 3rd and 7th days after the first miR-30e agomir treatment (Fig. 3b). Furthermore, traction test showed that MPTP caused a significant decrease in limb movements scored compared with saline group. However, miR-30e agomir delivery time-dependently increased the limb movements scored (Fig. 3c). Finally, MPTP significantly increased the time required for the mouse to cross the beam, whereas miR-30e upregulation decreased the latency time on the beam at 3rd and 7th days after the first miR-30e agomir treatment (Fig. 3d). Collectively, these data suggest that miR-30e overexpression can effectively improve the dyskinesia in PD mice model.

### MiR-30e attenuated dopaminergic neuronal loss and α-syn expression in SNpc of MPTP-PD mice

**Fig. 4:**
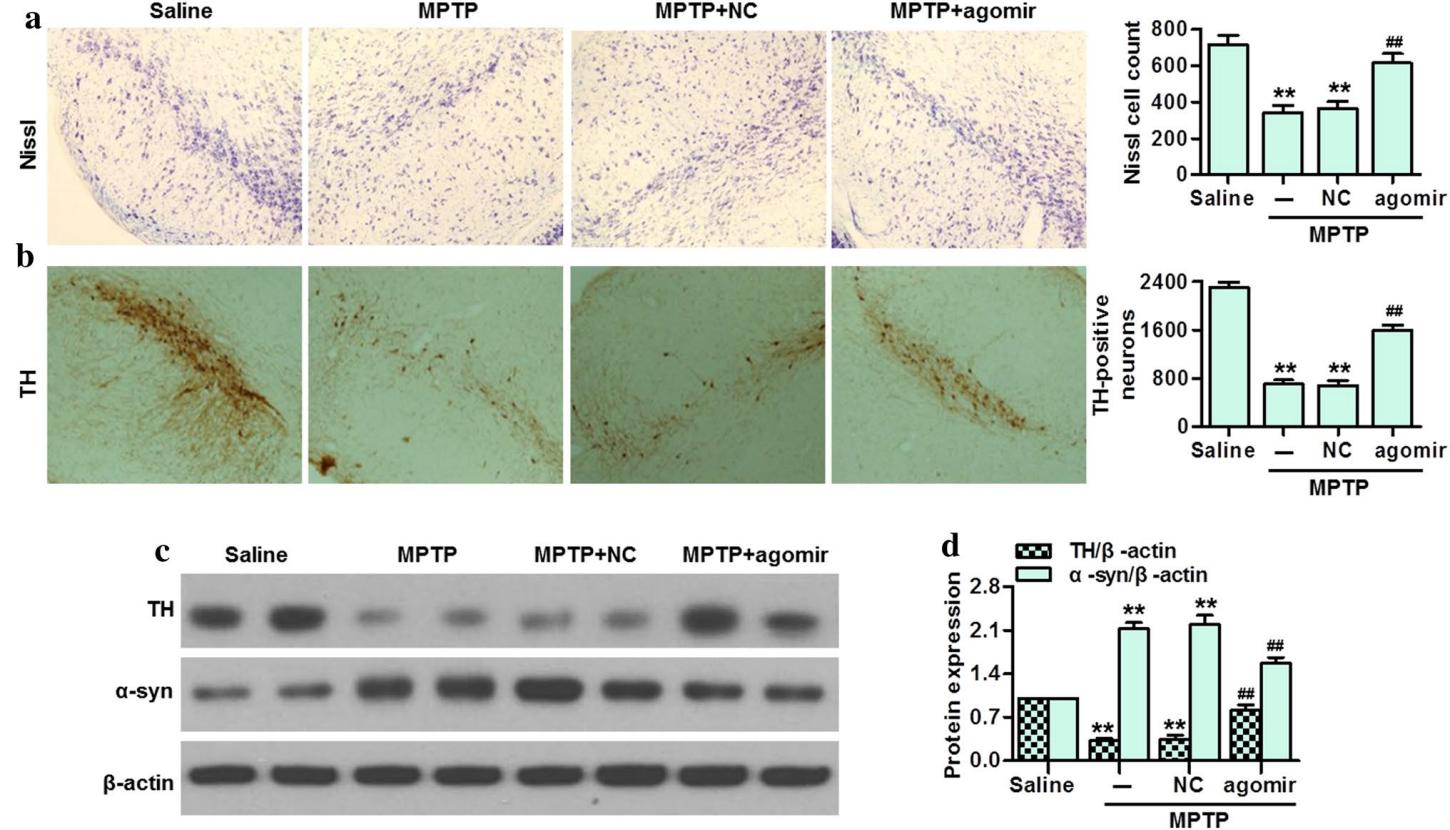
Exogenous delivery of miR-30e agomir protected against neuronal damage and dopaminergic neuronal loss in MPTP-induced PD mice model. **a** The nissl staining in SNpc. **b** Immunostaining of TH-positive neurons in the SNpc. **c** The protein expressions of TH and α-syn were determined by western blotting. **d** Densitometric analysis of TH and α-syn protein expression. **P < 0.01 vs. saline; ##*P* < 0.01 vs. MPTP, *n* = 6–8 mice in each group

To confirm the effect of miR-30e on neuronal activity, nissl staining was used to detect the level of nissl substance in SNpc. The results showed that the level of nissl substance was gradually decreased at 1st, 3rd and 7th days after the first MPTP injection (Figure S2). However, treatment with miR-30e agomir significantly restored the loss of nissl substance (Fig. 4a). We also determined the effect of miR-30e upregulation on dopaminergic neuronal loss in SNpc of MPTP-PD mice. MPTP resulted in a significant decrease in the number of TH-positive cells, and treatment with miR-30e markedly attenuated this TH loss in SNpc (Fig. 4b). Similarly, western blotting showed that upregulation of miR-30e could inhibit MPTP-induced decrease of TH protein expression. Moreover, we found that MPTP increased α-syn expression, whereas miR-30e agomir treatment was associated with decreased α-syn expression (Fig. 4c, d). These results suggest that miR-30e protects against MPTP-induced neuronal damage and dopaminergic neuronal loss.

### Effect of MiR-30e on inflammatory markers and BDNF levels in MPTP-PD mice

**Fig. 5:**
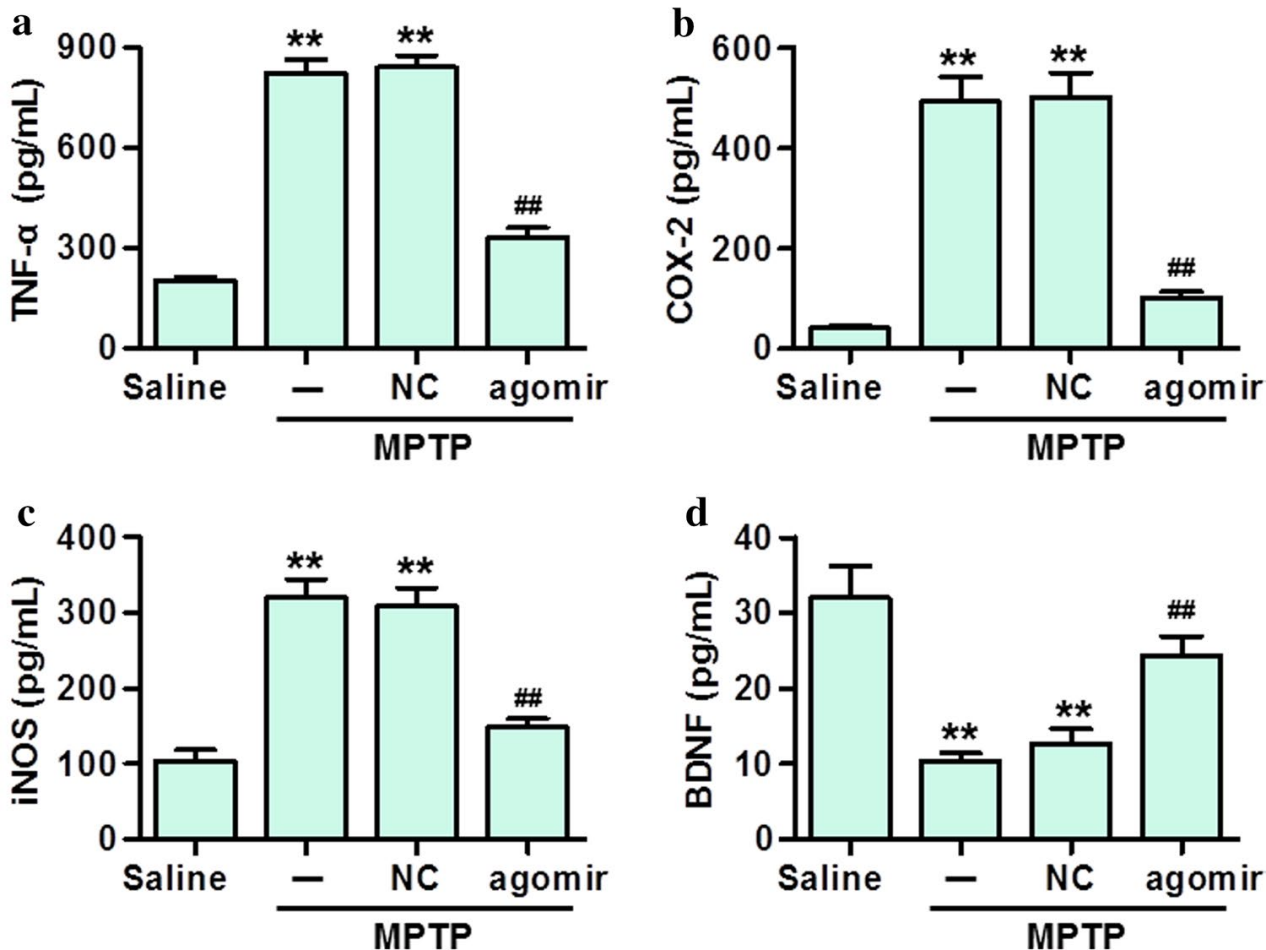
Effect MiR-30e agomir on inflammatory markers and BDNF levels in MPTP-PD mice. **a**–**d** The level of TNF-α (**a**), COX-2 (**b**), iNOS (**c**) and BDNF (**d**) in SNpc were determined using immunoassay kits. **P < 0.01 vs. saline; ##*P* < 0.01 vs. MPTP, *n* = 6 mice in each group

Since that α-syn-induced neuroinflammaiton has an important role in the pathogenesis of PD [19], we next detected the effect of miR-30e on inflammation in SNpc tissues. ELISA results showed that MPTP injection significantly increased the secretion of inflammatory mediators, TNF-α, COX-2 and iNOS. However, miR-30e treatment markedly suppressed the secretion of these inflammatory mediators induced by MPTP (Fig. 5a–c). In addition, the level of BDNF was significantly lower in the MPTP group than in saline group, and miR-30e agomir treatment could restore the decrease of BDNF level (Fig. 5d).

### Nlrp3 is a target gene of miR-30e

**Fig. 6:**
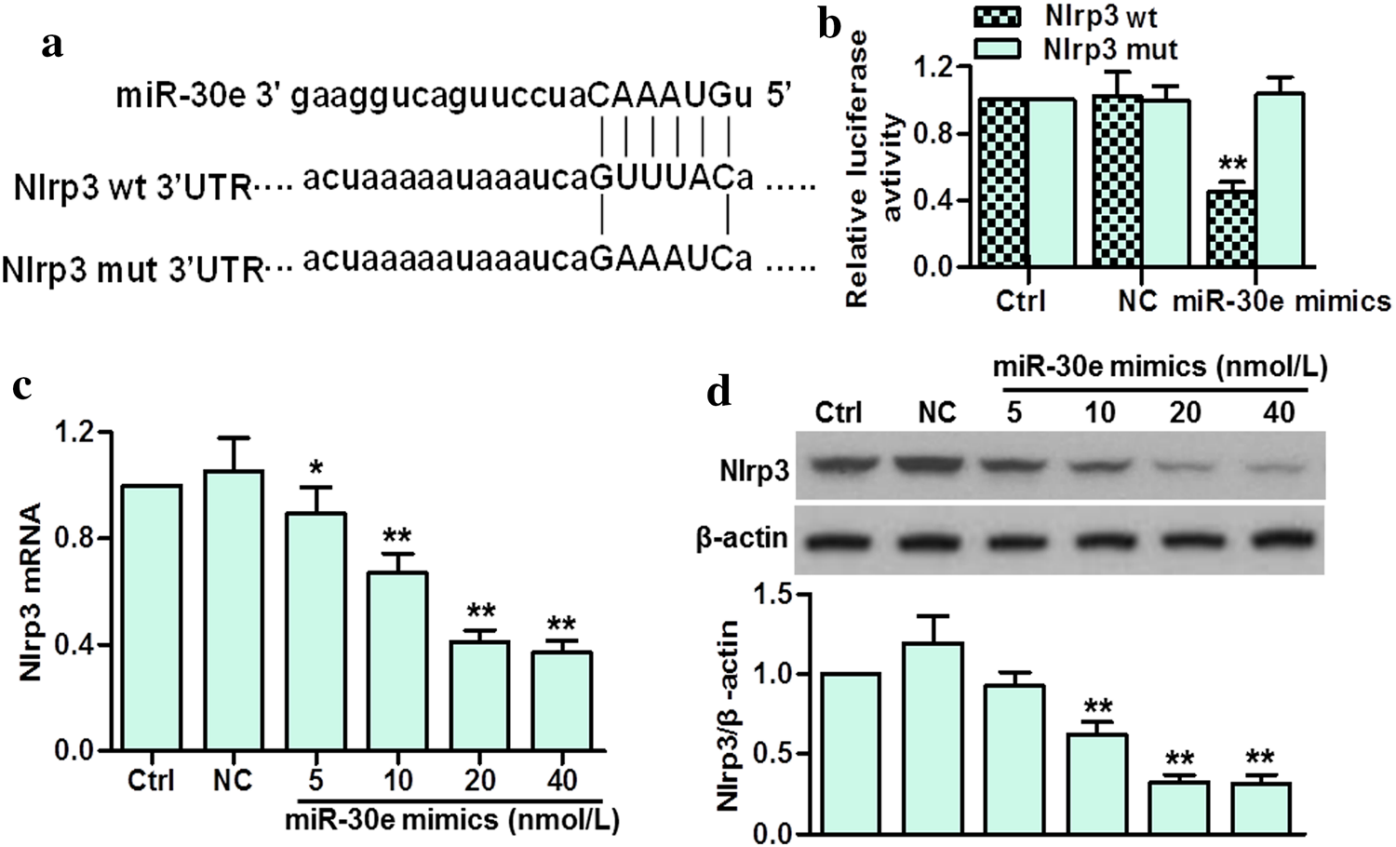
MiR-30e negatively regulated Nlrp3 expression. **a** Alignment of miR-30e binding site to Nlrp3 3′UTR was shown. **b** Luciferase activity in BV-2 cells transfected with wild-type or mutant Nlrp3 3′UTR reporter. **c** BV-2 cells were transfected with different concentrations of miR-30e mimics (5, 10, 20 or 40 nmol/L) for 48 h. Nlrp3 mRNA expression was determined by qRT-PCR. **d** Western blotting analysis of Nlrp3 in BV-2 cells transfected with miR-30e mimics or negative control. *P < 0.05, **P < 0.01 vs. control, *n* = 6

To explore the mechanisms underlying the neuroprotective effect of miR-30e, we analyzed the potential targets predicted for miR-30e. By using miRNA target gene prediction website (http://www.microrna.org/), we found that Nlrp3 was predicated as a putative target with a conserved miR-30e binding sites in its 3′UTR (Fig. 6a). When we co-transfected BV-2 cells with miR-30e mimics and wild-type or mutant Nlrp3 3′UTR reporter, luciferase assay showed that miR-30e overexpression significantly decreased the luciferase activity of wild-type Nlrp3 3′UTR reporter but not in the mutant one (Fig. 6b), suggesting that miR-30e directly binds the mRNA encoding Nlrp3. In consistence, transfection with miR-30e mimics dose-dependently decreased Nlrp3 mRNA expression (Fig. 6c). We also detected the effect of miR-30e mimics on endogenous Nlrp3 protein expression. Western blotting showed that miR-30e overexpression effectively decreased the protein expression of Nlrp3 (Fig. 6d).

### MiR-30e suppressed Nlrp3 inflammasome activation in SNpc of MPTP-PD mice

**Fig. 7:**
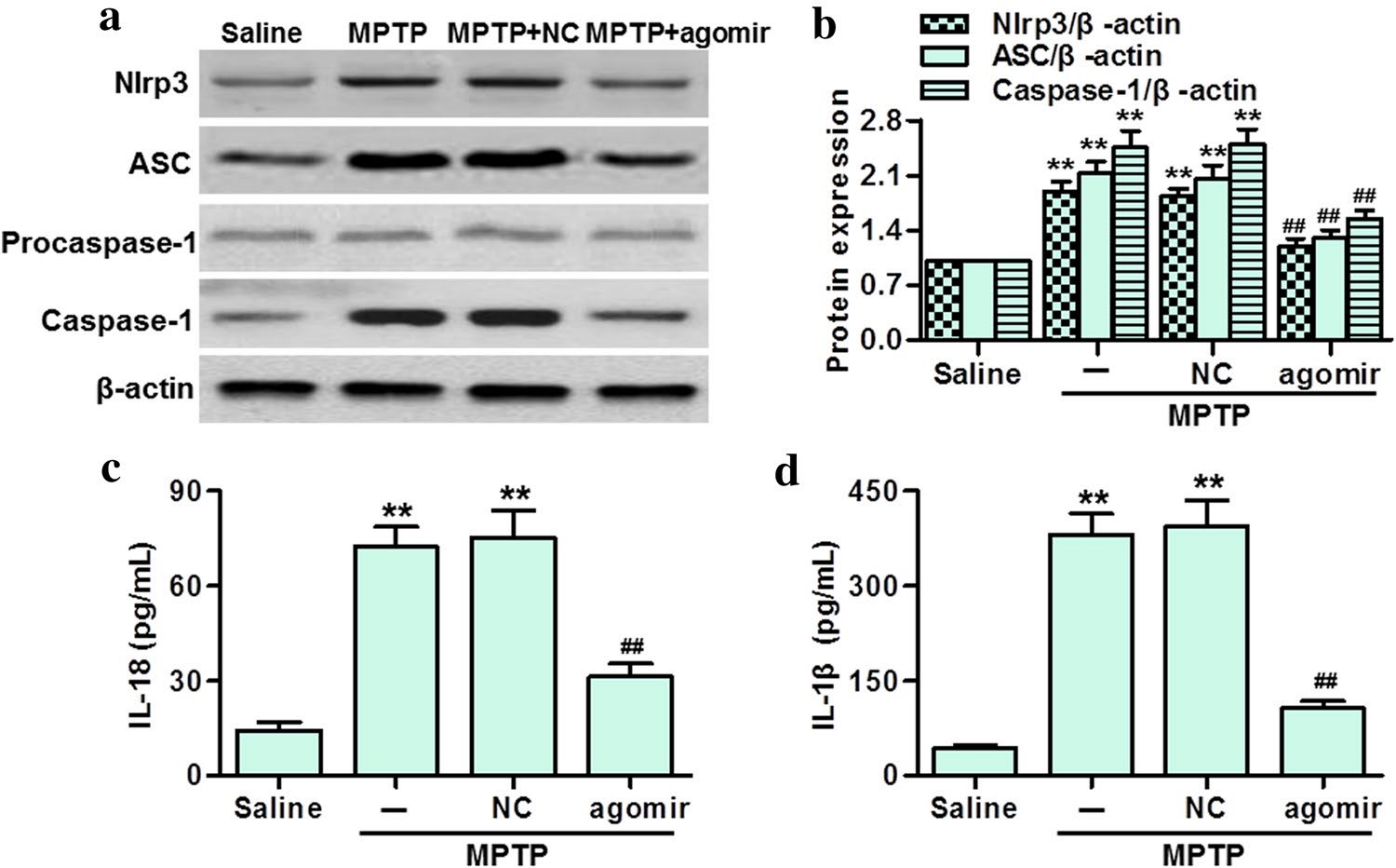
MiR-30e upregulation attenuated Nlrp3 inflammasome activation in SNpc of MPTP-PD mice. **a** Western blotting analysis of Nlrp3, ASC, Procaspase-1 and Caspase-1 protein expressions. **b** Densitometric analysis of the above genes protein expressions. **c**, **d** The level of IL-18 (**c**) and IL-1β (**d**) in SNpc were determined using immunoassay kits. **P < 0.01 vs. saline; ##*P* < 0.01 vs. MPTP, *n* = 5 mice in each group

Because of the crucial role of miR-30e in regulating Nlrp3 expression, we determined whether miR-30e controls the activity of Nlrp3 inflammasome in SNpc of MPTP-PD mice. Western blotting showed that the protein expressions of Nlrp3, ASC and Caspase-1 were significantly increased in MPTP-PD mice whereas the protein expression of Procaspase-1 was not altered. However, miR-30e upregulation abolished MPTP-induced increase of Nlrp3, ASC and Caspase-1 expressions (Fig. 7a, b). Moreover, the elevation of IL-18 and IL-1β secretions was markedly inhibited in MPTP-induced PD mice treated with miR-30e agomir (Fig. 7c, d). We also detected the mRNA levels of the Nlrp3 inflammasome. The results showed that MPTP injection significantly increased the mRNA expressions of Nlrp3, Caspase-1, ASC, IL-18 and IL-1β as compared with saline group. MiR-30e agomir treatment was associated with decreased expression of the above genes (Figure S3 A–E).

## Discussion

This study uncovers a link between miR-30e and Nlrp3 inflammasome-mediated neuroinflammation in the pathogenesis of PD. We provide convinced evidence that miR-30e improves neuronal damage, neuroinflammaiton and dyskinesia via negatively regulating Nlrp3 expression and inhibiting NLRP3 inflammasome activation in MPTP-induced PD mice model. MPTP is the most valuable neurotoxin for inducing animal PD model that producing many features of the biological and pathological changes similar to human PD [20]. After rapidly crossing the blood–brain barrier by systemic injection, MPTP is taken up by the astrocytes and catalyzed into the toxic moiety that can be transported into dopaminergic neurons, leading to neuronal damage and dyskinesia [21, 22]. Thus, here, we used MPTP to stimulate dopaminergic neuron loss in vivo to induce PD.

MiRs have been shown to act at the post-transcriptional level by binding the 3′UTR of their target mRNA, leading to degradation of the target gene expression [16]. To date, there are a few studies revealing the critical role of miRs in the pathogenesis of PD. For example, miR-133b expression was found to be decreased in the midbrain of PD patients as well as in mouse models [23]. Moreover, miR-124 targeted to bim and in turn inhibited dopaminergic neurons loss, a key event during the development of PD [17]. In addition, miR-7, miR-153 and miR-155 negatively regulated α-syn expression, which is a crucial regulator for neuroinflammation in PD [15, 24]. In the present study, we investigated the alteration of miR-30e in SNpc by qRT-PCR and the results showed that the expression of miR-30e was downregulated gradually after MPTP injection, suggesting miR-30 might also have a role in the pathogenesis of PD.

Although miR-30e has been shown to be involved in the regulation of glioma cells differentiation and invasion [25, 26], the exact role of miR-30e in PD has not been shown previously. As mentioned in the evidence cited above, MPTP administration is known to decrease neuronal activity and the density of TH-positive neurons, indicating degeneration of the dopaminergic neurons in SNpc [4, 8, 22]. The deficiency of dopamine level makes patients suffer from different degree of behavioral motor deficit [1]. In the current study, our results showed that MPTP injection produced behavior disorder, as evidenced by rota-rod test, pole test, traction test and beam-crossing task. However, delivery of miR-30e agomir in midbrain effectively prolonged the duration time of mice on rotating-stick, decreased the latency to cross straight run way on narrow beam, and increased the grasping force as well as the rate of climbing pole. Furthermore, we investigated whether miR-30e upregulation improves motor function through protecting against MPTP-induced neuronal damage. Nissl staining showed that restoration of miR-30e in PD mice could increase the neuronal activity. In addition, the loss of TH activity as well as a decrease in TH protein expression is thought to contribute to dopamine deficiency, which is the most prominent at media levels of SNpc [22]. Immunohistochemistry and western blotting analysis for TH expression revealed that the loss of dopamine neuron in PD mice was dramatically less pronounced after miR-30e agomir delivery. These results indicate that miR-30e can protect against neuronal injury in MPTP-induced PD mice model.

It has been reported that excessive accumulation of α-syn is a pathological hallmark of PD patients, especially in SNpc [19, 24]. Here, we demonstrated that miR-30e overexpression could effectively attenuate MPTP-induced the increase of α-syn expression in SNpc. Considering that α-syn-triggered neuroinflammation has an important in the pathogenesis of PD [6], we also examined the effect of miR-30e on inflammatory cytokines secretion in SNpc. The results showed that miR-30e upregulation almost abolished the increase of TNF-α, COX-2 and iNOS secretion. Moreover, aberrant alterations in BDNF expression or signaling may contribute to neurodegeneration and sustained decreased BDNF mRNA expression can be observed in SNpc of PD patients [27]. In this study, we found that the reduction of BNDF secretion in SNpc was markedly reversed by miR-30e agomir treatment.

Finally, we explored the mechanisms by which miR-30e inhibited neuroinflammation in SNpc of PD mice. Intriguing, although MPTP-induced α-syn expression was inhibited by miR-30e agomir, we found that the luciferase activity of α-syn was not affected by miR-30e (data not shown), suggesting α-syn is not the direct target of miR-30e. Notably, α-syn has been recognized to induce the IL-1β production in a process that depends, at least partially, on Nlrp3 inflammasome [6, 28]. In the current study, we demonstrated for the first time that Nlrp3 was a potential target of miR-30e. The luciferase assay indicated that miR-30e targeted the 3′UTR region of Nlrp3 to negatively regulate Nlrp3 mRNA and protein expression. In response to a variety of inflammatory stimuli, the Nlrp3 inflammasome, along with the adaptor protein ASC, induces the activation of Caspase-1 and the maturation of proinflammatory cytokines IL-18 and IL-1β, leading to trigger inflammation [11, 29]. Accordingly, our results showed that Nlrp3, ASC and Caspase-1 expressions, and IL-18 and IL-1β secretions were all increased in SNpc of PD mice. However, miR-30e restoration abolished the above elevations. Consistent with the protein expressions in SNpc, the mRNA levels of the Nlrp3 inflammasome were also decreased after miR-30e agomir treatment. These data suggest that the activation of Nlrp3 inflammasome may contribute to MPTP-induced neuroinflammation in SNpc, whereas miR-30e inhibits this process by targeting Nlrp3. Furthermore, considering the critical role of Nlrp3 inflammasome in the development of neurodegenerative diseases [6, 12, 13], our study also indicate that miR-30e induces neuron regeneration at least partially via inhibition Nlrp3 inflammasome-mediated inflammation.

In conclusion, our study demonstrates that miR-30e negatively regulates Nlrp3 expression, which in turn attenuates neuroinflammation in SNpc of PD mice through inhibiting Nlrp3 inflammasome activity. These findings indicate that targeting miR-30e by a genetic approach may provide a novel strategy for the treatment of PD.

## Electronic supplementary material

Below is the link to the electronic supplementary material.

Supplementary material 1 (DOC 135 kb)
