# Supplementary material for: MicroRNA-30e regulates neuroinflammation in MPTP model of Parkinson’s disease by targeting Nlrp3
Source: Hum Cell. 2017 Dec 22;31(2):106–15. doi: 10.1007/s13577-017-0187-5 (PMC5852205; doi:10.1007/s13577-017-0187-5)
Supplement: Supplementary file 1 — Supplementary material 1 (DOC 135 kb) [file 13577_2017_187_MOESM1_ESM.doc]

**MicroRNA-30e regulates neuroinflammation in MPTP model of Parkinson’s disease by targeting Nlrp3**

Dongsheng Li^a^, Hongqi Yang^a^, Jianjun Ma^a^, Sha Luo^a^, Siyuan Chen^a^, Qi Gu^a^

^a^Department of Neurology, Henan Provincial People’s Hospital, Zhengzhou, Henan, 450003, China

**Running title:** MicroRNA-30e regulates neuroinflammation

**Please address inquiries to:** Dongsheng Li, Department of Neurology, Henan Provincial People Hospital, NO. 7 Weiwu Road, Jinshui District, Zhengzhou, Henan, 450003, China. Tel: +86 0371 65367922. Fax: +86 0371 65367922. E-mail: dongshengli2014@163.com

**Disclosure of conflict of interest**

None.

**Supplemental files**

**Table S1. The primer sequences for qRT-PCR**

| Genes | | Primers sequences | |
| --- | --- | --- | --- |
| *MiR-30e* | Forward | | 5’-GGGCAGTCTTTGCTACTGTAAAC-3’ |
|  | Reverse | | 5’-GCCGCTGTAAACATCCGACT-3’ |
| *Nlrp3* | Forward | | 5’-AGCTGCTCTTTGAGCCTGAG-3’ |
|  | Reverse | | 5’-CTTGCACACTGGTGGGTTTG-3’ |
| *Caspase-1* | Forward | | 5’-GACCGAGTGGTTCCCTCAAG-3’ |
|  | Reverse | | 5’-GACGTGTACGAGTGGGTGTT-3’ |
| *ASC* | Forward | | 5’-GAAGCTGCTGACAGTGCAAC-3’ |
|  | Reverse | | 5’-AGGAGGAACAGTTAAGCGCC-3’ |
| *IL-18* | Forward | | 5’-ACGTGTTCCAGGACACAACA-3’ |
|  | Reverse | | 5’-GGCGCATGTGTGCTAATCAT-3’ |
| *IL-1β* | Forward | | 5’-CCTTGTCGAATGGGCAGT-3’ |
|  | Reverse | | 5’-CAGGGAGGGAAACACACGTT-3’ |
| *GAPDH* | Forward | | 5’-GGTTGTCTCCTGCGACTTCA-3’ |
|  | Reverse | | 5’-CCCTAGGCCCCTCCTGTTAT-3’ |

**Supplementary Figures**

**Figure S1**


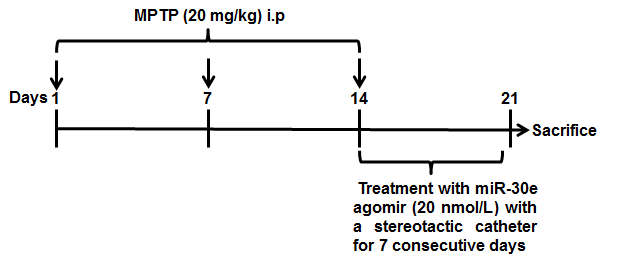


**Figure S1. Study protocol design.** The 8-week-old male C57BL/6 mice were received 3 times of intraperitoneally (i.p.) injection of MPTP (20 mg/kg) at day 1, 7, and 14. MiR-30e agomir was injected through the catheter per day for 7 consecutive days.

**Figure S2**

**
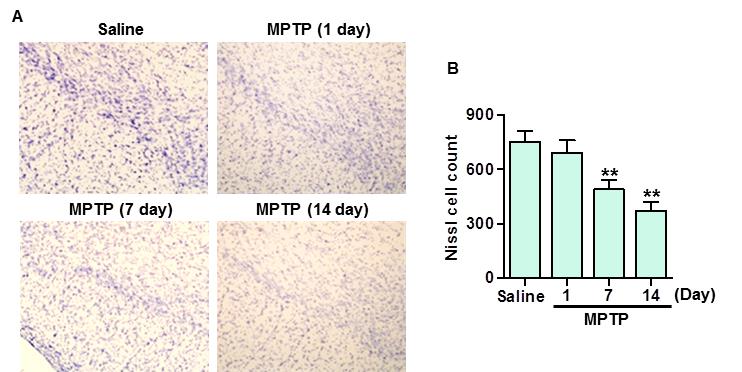
**

**Figure S2. Loss of nissl substance in SNpc after MPTP injection.** (A) Mice were killed at 1, 7, and 14 days after the first MPTP injection. Representative images of nissl staining in SNpc. (B) Stereological cell quantification of nissl cells. **P<0.01 vs. saline, n=6 mice in each group.

**Figure S3**


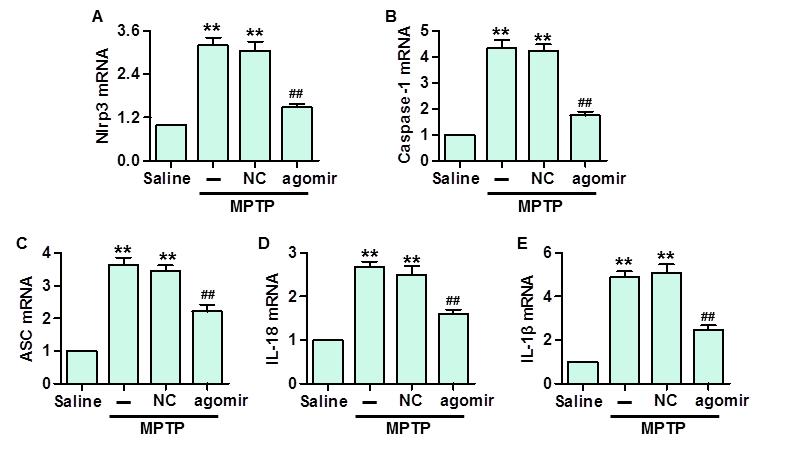


**Figure S3. MiR-30e decreased the activity of Nlrp3 inflammasome in SNpc of MPTP-PD mice.** (A-E) QRT-PCR analysis of the mRNA expression of Nlrp3 (A), Caspase-1 (B), ASC (C), IL-18 (D) and IL-1β (E) in SNpc. **P<0.01 vs. saline; ##P<0.01 vs. MPTP, n=6 mice in each group.
